# Characteristics of Gut Microbiota in Patients with Chronic Obstructive Pulmonary Disease Based on Metagenomics and Metabolomics

**DOI:** 10.3390/ijms27104213

**Published:** 2026-05-09

**Authors:** Yanan Wang, Xiaoyan Liu, Ruiyue Gao, Yu An, Chao Ren, Li An

**Affiliations:** 1 Department of Respiratory and Critical Care Medicine, Beijing Institute of Respiratory Medicine, Beijing Chao-Yang Hospital, Capital Medical University, Beijing 100020, China; wyn960202@sina.com (Y.W.);; 2 Beijing Key Laboratory of Multimodal Intelligent Diagnosis and Treatment System for Respiratory Diseases, Beijing Chao-Yang Hospital, Capital Medical University, Beijing 100020, China; lxycyyy@mail.ccmu.edu.com (X.L.);; 3 Medical Research Center, Beijing Institute of Respiratory Medicine, Beijing Chao-Yang Hospital, Capital Medical University, Beijing 100020, China

**Keywords:** chronic obstructive pulmonary disease, gut microbiota, metagenomic sequencing, untargeted metabolomics, riboflavin

## Abstract

The gut–lung axis is important in Chronic Obstructive Pulmonary Disease (COPD) pathogenesis; however, most studies rely on low-resolution 16S rRNA sequencing, and integrated multi-omics investigations in Chinese COPD populations are scarce. A total of 104 participants including 74 stable COPD patients and 30 healthy controls from northern China were recruited, and shotgun metagenomic sequencing and untargeted metabolomics were performed. Results showed that alpha diversity of the gut microbiota did not differ significantly between COPD patients and healthy controls, whereas beta diversity showed clear separation. Marked differences in microbial composition from phylum to species levels (e.g., *Oscillospiraceae*) and altered microbial functions (signal transduction, antibiotic resistance, etc.) were observed in COPD patients. Metabolomic profiling identified 497 differential fecal metabolites and 1260 differential serum metabolites in COPD patients. Importantly, serum riboflavin levels were significantly reduced and positively correlated with pulmonary function indices as well as the key differential gut microbial functional gene K11752. Serum metabolite eremopetasinorol exhibited high diagnostic accuracy for COPD (AUC = 0.947, 95% CI: 0.8–0.98), surpassing fecal metabolites and microbial features. This study provides integrated metagenomic and metabolomic characterization of gut microbiota alterations in Chinese COPD patients, offering novel insights for biomarker discovery and targeted intervention strategies.

## 1. Introduction

Chronic obstructive pulmonary disease (COPD) is a progressive and largely irreversible chronic airway disease that affects approximately 400 million individuals worldwide and currently ranks as the third leading cause of global mortality [1,2]. Despite its high prevalence and disease burden, early-stage COPD is frequently underdiagnosed because its initial clinical manifestations are often subtle and nonspecific [3]. Spirometry remains the diagnostic gold standard; however, its routine implementation in clinical practice is limited by technical complexity and accessibility constraints. As a result, many patients are diagnosed at advanced stages, when therapeutic options are less effective, leading to impaired quality of life and unfavorable long-term outcomes [4,5]. These challenges underscore the urgent need to identify novel biomarkers and therapeutic targets that enable earlier detection and more precise disease management.

The gut microbiome constitutes the largest and most extensively studied microbial ecosystem in the human body [6,7]. Increasing evidence supports a bidirectional interaction between the gut microbiota and pulmonary health, a relationship conceptualized as the “gut–lung axis”. This framework describes dynamic communication between the gastrointestinal tract and the lungs mediated through shared mucosal immune networks, systemic circulation, and microbiota-derived metabolites. Within this axis, alterations in gut microbial composition and function can influence pulmonary immunity and inflammation, while lung pathology can, in turn, disrupt intestinal microbial homeostasis [8,9].

Substantial progress has been made in elucidating the role of gut microbiota and microbial metabolites in COPD. Using 16S rRNA sequencing, Bowerman et al. reported a significant reduction in gut microbial diversity in COPD patients, accompanied by alterations in metabolite profiles, including decreased levels of anti-inflammatory short-chain fatty acids (SCFAs) [10]. In parallel, reductions in beneficial bacterial genera such as *Bifidobacterium* and *Prevotella*, together with increased abundance of potentially pathogenic taxa including *Streptococcus* and *Escherichia-Shigella*, have been observed, suggesting a link between gut dysbiosis and systemic inflammation in COPD [11]. Additional studies demonstrated that patients experiencing acute exacerbations of COPD exhibit further reductions in gut microbial alpha diversity and SCFA abundance compared with those in the stable phase [12,13]. Moreover, Mendelian randomization analysis conducted by Cao et al. identified a positive causal association between the microbial metabolite phenylacetylglutamine and COPD, highlighting its potential relevance as a therapeutic target [14]. Collectively, these findings indicate that gut microbiota and their metabolites play an important regulatory role in COPD pathogenesis and disease progression.

However, the majority of prior investigations relied on 16S rRNA sequencing, which provides limited taxonomic resolution and does not allow comprehensive assessment of microbial functional capacity or metabolite-related pathways. Although a metagenomic-based study attempted to address these limitations, it was constrained by a small sample size drawn from an Australian cohort [10], thereby limiting the representativeness and generalizability of its conclusions. Therefore, integrated analyses incorporating metagenomics and metabolomics, particularly in Chinese populations, remain scarce and warrant further exploration.

In this context, the present study employed shotgun metagenomic sequencing to systematically characterize the compositional and functional features of the gut microbiota in patients with stable COPD from northern China. By comparing individuals with normal pulmonary function to those with COPD, the study aimed to elucidate the regulatory role of gut microbiota in COPD pathogenesis. In parallel, untargeted metabolomics was integrated to identify metabolic alterations and potential biomarkers associated with disease development and progression. Together, these analyses aim to provide a scientific foundation for improved clinical prediction, early diagnosis, and targeted intervention strategies for COPD.

## 2. Results

### 2.1. Baseline Characteristics of the Study Population

A total of 74 participants with COPD and 30 healthy control subjects were included in the analysis. Among patients with COPD, 11 were classified as Global Initiative for Chronic Obstructive Lung Disease (GOLD) stage I, 33 as GOLD stage II, 19 as GOLD stage III, and 10 as GOLD stage IV. The demographic and clinical characteristics of all participants are presented in Table 1. There was no statistically significant difference in age between the COPD and control groups (*p* > 0.05). In contrast, the COPD group demonstrated significantly lower Body Mass Index (BMI), measured Forced Vital Capacity (FVC), FVC% predicted, measured Forced Expiratory Volume in 1 second (FEV_1_), FEV_1_% predicted, and FEV_1_/FVC ratio compared with healthy controls (*p* < 0.05). In addition, both the prevalence of smoking history and cumulative smoking exposure, expressed as pack-years, were significantly higher in the COPD group than in the control group (*p* < 0.05).

### 2.2. Gut Microbiota Composition in Patients with COPD

**Figure 1:**
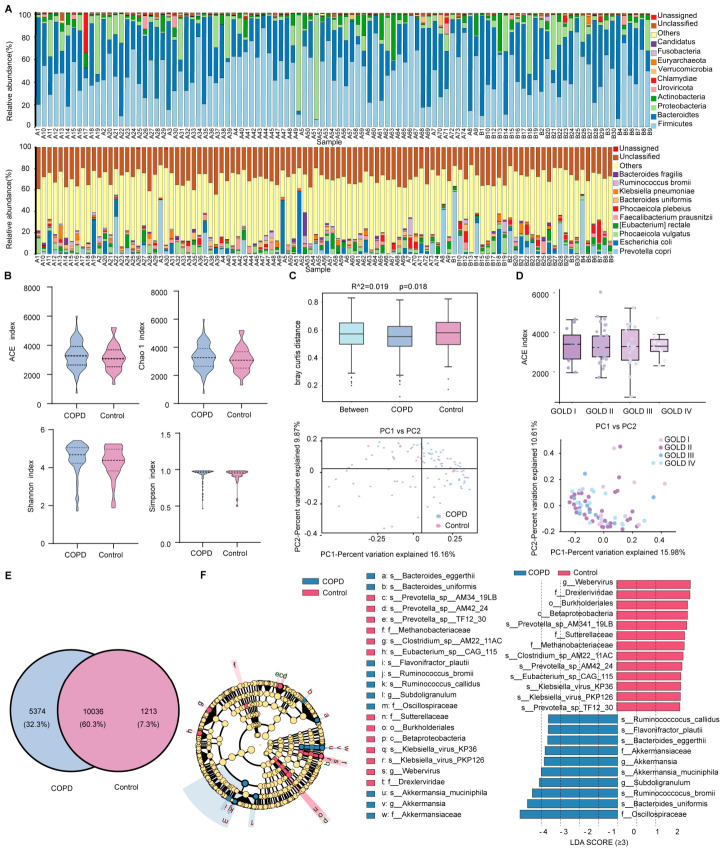
Gut microbiota composition and diversity in fecal samples from patients with COPD and healthy controls based on metagenomic sequencing. (**A**) Bar plot depicting the overall gut microbiota composition across all fecal samples, with each color representing a microbial species and bar length indicating relative abundance. The distribution of the top 13 most abundant taxa is shown at the phylum and species levels. (**B**) Comparison of gut microbiota alpha diversity between the COPD and control groups, assessed using the ACE, Chao1, Shannon, and Simpson indices. (**C**) PCoA based on Bray–Curtis distances and permutational multivariate analysis of variance (PerMANOVA) evaluating differences in gut microbiota beta diversity between the two groups. (**D**) Alpha and beta diversity analyses of gut microbiota following stratification of COPD patients by GOLD grade. (**E**) Venn diagram illustrating the number of shared and unique microbial species between the COPD and control groups at the species level. (**F**) Cladogram showing differentially abundant gut microbial taxa and corresponding bar plot of LDA scores derived from LEfSe analysis.

Metagenomic sequencing was used to characterize the gut microbiota composition in fecal samples from COPD patients and healthy controls. At the phylum level, the gut microbiota of both groups was predominantly composed of *Firmicutes* and *Bacteroidetes*. At the class level, *Clostridia* and *Bacteroidia* exhibited the highest relative abundances in both cohorts. At the order level, *Eubacteriales* and *Bacteroidales* were the dominant taxa in the COPD group, whereas *Bacteroidales* predominated in the control group. At the family level, *Bacteroidaceae* and *Lachnospiraceae* constituted the major components of the gut microbiota in both groups. At the genus level, *Bacteroides* and *Ruminococcus* were the most abundant taxa in the COPD group, whereas *Prevotella* and *Bacteroides* showed the highest relative abundances in healthy controls. At the species level, the gut microbiota of COPD patients was primarily characterized by *Escherichia coli* and *Prevotella copri*, while *Prevotella copri* and *Phocaeicola vulgatus* were the dominant species in the control group (Figure 1A and Figures S1–S3).

### 2.3. Gut Microbiota Diversity in Patients with COPD

To evaluate differences in gut microbiota diversity between patients with COPD and healthy controls, alpha diversity indices were first assessed. As shown in Figure 1B, no significant differences were observed between the two groups in the ACE index, Chao1 index, Shannon index, or Simpson index. In contrast, beta diversity analysis based on Bray–Curtis distance matrices demonstrated a significant separation in overall gut microbiota composition between the COPD group and healthy controls (*p* = 0.018), as visualized by Principal coordinates analysis (PCoA) (Figure 1C). In addition, stratified analysis revealed no significant differences in gut microbiota diversity among patients with COPD across GOLD grades I to IV (Figure 1D). Accordingly, subsequent analyses were conducted by comparing the COPD group as a whole with the control group, without further stratification by disease severity.

### 2.4. Differential Gut Microbiota Composition in Patients with COPD

To further characterize differences in gut microbiota composition associated with COPD, bacterial taxa were compared between the COPD and control groups across multiple taxonomic levels. Venn diagram analysis at the species level showed that the two groups shared 10,036 bacterial species. The COPD group harbored 5374 unique species, whereas 1213 species were unique to the healthy control group, indicating substantial divergence in species composition between the groups (Figure 1E).

Linear discriminant analysis Effect Size (LEfSe) was subsequently applied to identify taxa with significantly different relative abundances between groups (Figure 1F). This analysis identified 23 taxa with differential abundance in the COPD group, using an linear discriminant analysis (LDA) score threshold of ≥3, highlighting distinct gut microbiota signatures associated with COPD. Taxa including *g_Subdoligranulum*, *s_Akkermansia muciniphila,* and *s_Flavonifractor plautii* were significantly enriched in the COPD group. In contrast, healthy controls exhibited higher relative abundances of taxa such as *f_Sutterellaceae*, *s_Eubacterium_sp_CAG_115*, *s_Preyotella_sp_AM42_24*, and *s_Preyotella_sp_TF12_30*, which have been reported in prior studies as beneficial bacteria associated with anti-inflammatory effects and maintenance of intestinal barrier integrity. Detailed characterization of these differentially abundant taxa may provide novel insights into potential diagnostic and therapeutic targets for COPD.

### 2.5. Functional Gene Profiling of the Gut Microbiota in Patients with COPD

**Figure 2:**
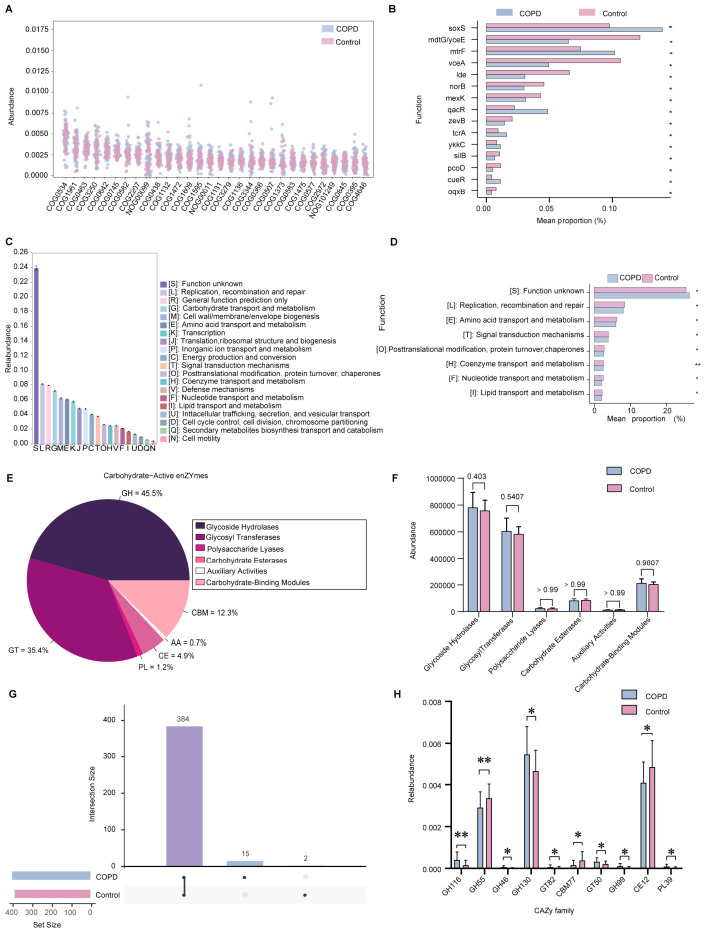
Functional gene profiles and carbohydrate-active enzyme composition of the gut microbiota in COPD and control groups. (**A**) Scatter plot showing the relative abundance of the top 30 functional genes across all samples. (**B**) Bar plot displaying the 15 functional genes with the most significant differences in abundance between the COPD and control groups. (**C**) Sorted bar chart illustrating the relative abundance of eggNOG functional categories across all samples. (**D**) Comparative analysis of eggNOG functional category abundance between the COPD and control groups. (**E**) Proportional distribution of annotated CAZy classes in the gut microbiota of the two groups. (**F**) Differential abundance analysis of carbohydrate-active enzyme classes between the COPD and control groups. (**G**) UpSet plot depicting shared and group-specific carbohydrate-active enzymes (The vertical line in the plot indicates the number of shared genes present in both the COPD and control groups. The individual dots represent the number of group-specific genes that are unique to either the COPD or control group and not found in the other.). (**H**) Bar chart of the top 10 carbohydrate-active enzymes with the most significant abundance differences between groups, ranked by statistical significance. (* *p* < 0.05, ** *p* ≤ 0.01). Note: The single-letter abbreviations used in Figure 2C,D denote the standard functional categories defined by the COG database.

To characterize functional alterations in the gut microbiota of patients with COPD, gene composition and functional prediction analyses were performed based on Evolutionary genealogy of genes: Non-supervised Orthologous Groups (eggNOG) annotation (Figure 2A). Across all samples, a total of 47,862 functional genes were identified, among which 2698 genes exhibited significant differences in abundance between the COPD and control groups. The top 15 functional genes demonstrating the most pronounced differences in relative abundance are shown in Figure 2B. Compared with healthy controls, the COPD group displayed significantly higher abundances of genes including *copS*, *vexH*, *chrA1*, *soxS*, and *mtrF*, whereas genes such as *ruvB*, *vceA*, *purH*, *yvdB*, and *dauA* were relatively depleted.

Functional categorization based on eggNOG annotation revealed that genes across both groups were distributed among 25 functional gene families. Among these categories, [S]: Function unknown accounted for the largest proportion of annotated genes (26.08%), while [Y]: Nuclear structure represented the smallest fraction (0.000018%) (Figure 2C). Comparative analysis of functional category abundance demonstrated that the COPD group exhibited significant enrichment of gene families associated with [T]: Signal transduction mechanisms and [S]: Function unknown. In contrast, healthy controls showed significantly higher representation of functional categories related to [O]: Posttranslational modification, protein turnover, chaperones, and [H]: Coenzyme transport and metabolism (Figure 2D).

### 2.6. Differential Analysis of Carbohydrate-Active Enzymes

To further explore potential alterations in carbohydrate metabolism associated with gut microbiota dysbiosis in COPD, carbohydrate-active enzyme profiles were analyzed using annotations derived from the Carbohydrate-Active enZYmes (CAZy) database. All samples were classified into six major carbohydrate-active enzymes (Figure S4). The overall distribution comprised glycoside hydrolases (45.5%), glycosyl transferases (35.4%), carbohydrate-binding modules (12.3%), carbohydrate esterases (4.9%), polysaccharide lyases (1.2%), and auxiliary activities (0.7%) (Figure 2E). No statistically significant differences were observed in the relative abundance of these six major enzyme classes between the COPD and control groups (Figure 2F). Despite the similarity at the class level, UpSet analysis revealed notable differences in the composition of carbohydrate-active enzyme subtypes between groups. Specifically, 15 carbohydrate-active enzymes were unique to the COPD group, two were unique to the control group, and 384 enzymes were shared between both groups (Figure 2G). Further differential abundance analysis identified 53 carbohydrate-active enzymes with significantly altered abundances between the two groups, with GH116 exhibiting the most pronounced difference (Figure 2H). Notably, the four carbohydrate-active enzymes with the largest abundance differences all belonged to the glycoside hydrolase family. These findings suggest that dysregulation of glycoside hydrolases may contribute to gut ecological imbalance in COPD and could be associated with alterations in digestive and metabolic function in affected individuals.

### 2.7. Antibiotic Resistance Gene Profiling

Antibiotic resistance represents a critical functional attribute of the gut microbiota and may influence disease pathogenesis, progression, and therapeutic responsiveness in patients with COPD. Accordingly, antibiotic resistance genes (ARGs) and resistance profiles were systematically analyzed by aligning metagenomic sequences against the Comprehensive Antibiotic Resistance Database (CARD). Across all samples, a total of 128,640 ARGs were identified. Differential abundance analysis revealed 70 ARGs with statistically significant differences between the COPD and control groups.

**Figure 3:**
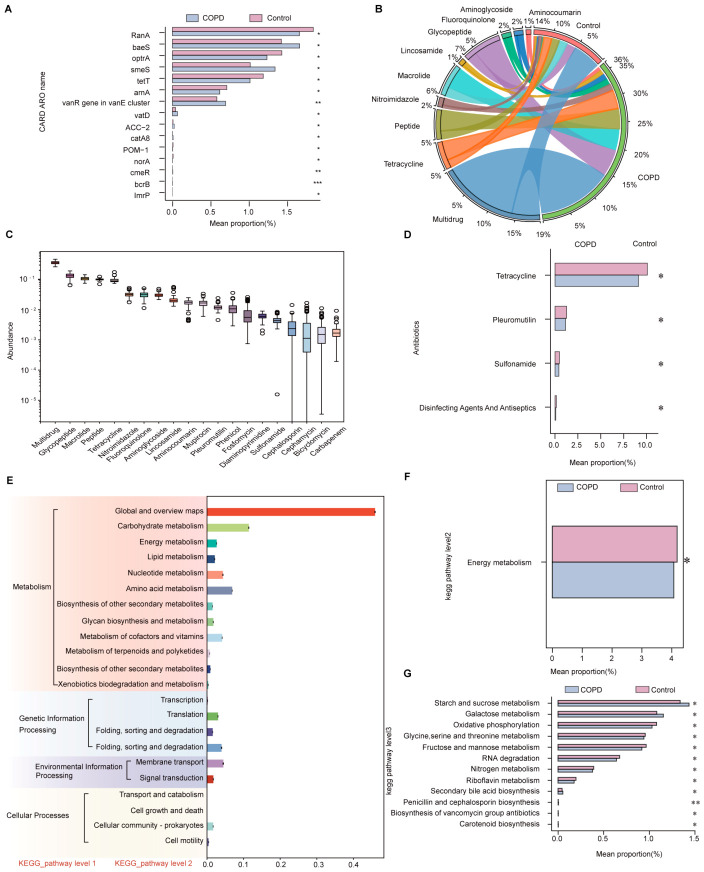
Antibiotic resistance gene profiles and metabolic pathway analyses in COPD and control groups. (**A**) Bar plot showing the top 15 antibiotic resistance genes with the most significant differences in relative abundance between the COPD and control groups. (**B**) Chord diagram illustrating the composition of antibiotic resistance genes annotated to the CARD database in both groups, highlighting the distribution of resistance gene types between COPD patients and healthy controls. (**C**) Top 20 antibiotic resistance mechanisms ranked by relative abundance across all samples. (**D**) Comparative analysis of resistance to different antibiotic classes between the COPD and control groups. (**E**) Hierarchical diagram depicting relationships between primary and secondary metabolic pathways. (**F**) Differential abundance analysis of secondary metabolic pathways between the COPD and control groups. (**G**) Differential abundance analysis of tertiary metabolic pathways between the two groups (* *p* < 0.05, ** *p* ≤ 0.01, *** *p* ≤ 0.001). Note: In the chord diagram, the outer ring is divided into two sections: the right side represents sample groups, and the left half represents annotated antibiotic resistance gene types. The scale reflects relative abundance. Ribbons within the inner circle link resistance gene types to sample groups, with ribbon width corresponding to the proportional contribution.

Several resistance-associated genes were significantly enriched in the COPD group, including *baeS* (ARO:3000829), *smeS* (ARO:3003067), the *VanR* gene within the VanE cluster (ARO:3002924), *VatD* (ARO:3002843), *Acc-2* (ARO:3001816), *catA8* (ARO:3004658), *cmeR* (ARO:3000526), and *bcrB* (ARO:3002988). In contrast, genes such as *RanA* (ARO:3005091), *optrA* (ARO:3003746), *tetT* (ARO:3000193), *arnA* (ARO:3002985), *POM-1* (ARO:3006974), *norA* (ARO:3000391), and *ImrP* (ARO:3003969) were significantly more abundant in the control group, indicating distinct resistance gene profiles between the two cohorts (Figure 3A).

At the antibiotic class level, differential resistance patterns were observed between groups, encompassing resistance to glycopeptides, macrolides, and tetracyclines (Figure 3B). The top 20 resistance mechanisms by relative abundance are shown in Figure 3C, with multidrug resistance constituting the most prevalent category across all samples. Compared with healthy controls, COPD patients exhibited significantly higher relative abundances of resistance to pleuromutilin and sulfonamide antibiotics, whereas resistance associated with tetracyclines and disinfecting agents and antiseptics showed a decreasing trend (Figure 3D).

### 2.8. Analysis of Microbial Metabolic and Signaling Pathways

Comparative pathway analysis based on functional annotation identified four major primary pathway categories across all samples: Cellular Processes (5.54%), Environmental Information Processing (15.85%), Genetic Information Processing (18.68%), and Metabolism (59.93%). Among these, Metabolism was the most abundant primary pathway category, whereas Cellular Processes accounted for the smallest proportion (Figure 3E). At the secondary pathway level, 22 metabolic pathways were identified in both groups. Within the Metabolism category, 12 secondary pathways were detected, including Amino Acid Metabolism and Energy Metabolism. Genetic Information Processing comprised four secondary pathways, such as Transcription and Translation. Environmental Information Processing included two pathways: namely Membrane Transport and Signal Transduction, while Cellular Processes encompassed four pathways, including Transport and Catabolism and Cellular Community–Prokaryotes. Among all secondary pathways, Global and Overview Maps exhibited the highest relative abundance (30.66%), whereas Cell Growth and Death showed the lowest relative abundance (0.0014%) (Figure 3E).

Rank-sum testing revealed a significant difference between the COPD and control groups in the secondary pathway of Energy Metabolism, which was significantly reduced in the COPD group (Figure 3F). Further analysis at the tertiary pathway level identified 172 distinct pathways. Pathways with relatively higher abundance in the COPD group included Starch and sucrose metabolism, Galactose metabolism, and Secondary bile acid biosynthesis. In contrast, Oxidative phosphorylation, RNA degradation, and Glycine, serine and threonine metabolism exhibited relatively lower abundances in COPD patients compared with controls (Figure 3G). Collectively, these findings highlight a complex reprogramming of gut microbial metabolic and signaling pathways in COPD, which may contribute to disease onset and progression.

### 2.9. Fecal Metabolomic Characteristics and Functional Alterations Associated with COPD

**Figure 4:**
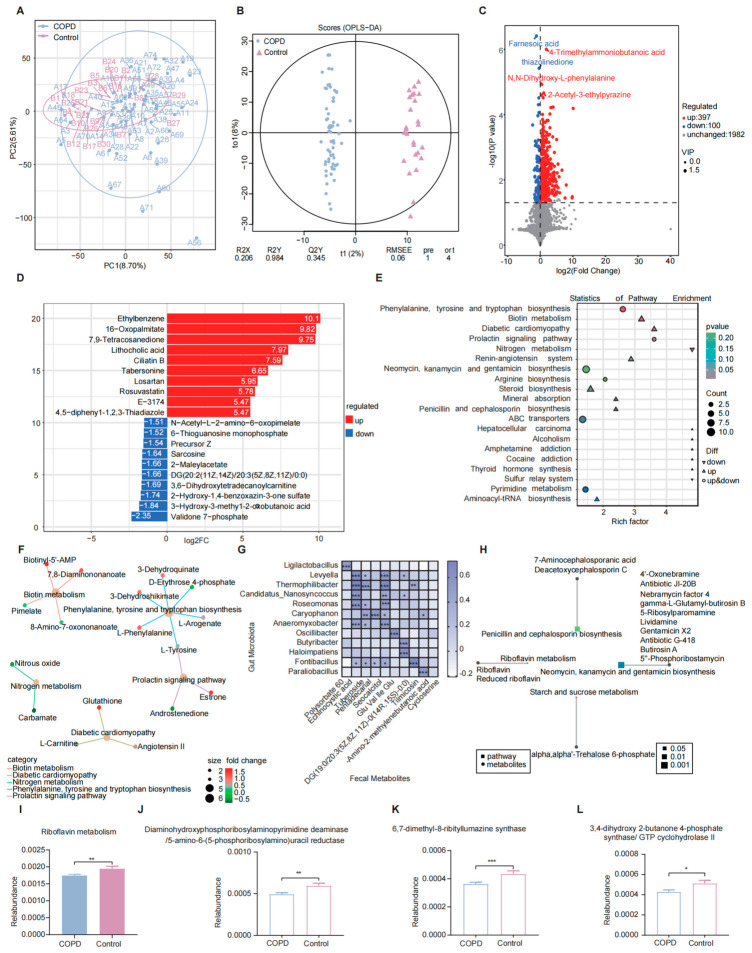
Fecal metabolomic profiling in COPD patients and integrated analysis with metagenomic data. (**A**) PCA score plot of fecal metabolite profiles. (**B**) OPLS-DA score plot and corresponding permutation validation plot; models with Q^2^Y > 0.5 were considered valid. (**C**) Volcano plot depicting differential metabolites identified between the COPD and control groups. (**D**) Bar plots showing the top 10 metabolites with the greatest fold decreases in the COPD group compared with controls. (**E**) KEGG pathway enrichment dot plot of differential metabolites. (**F**) Network diagram illustrating associations between differential metabolites and significantly enriched metabolic pathways. (**G**) Heatmap of Spearman correlation analysis between selected differential metabolites and selected differentially abundant microbial species (white indicates negative correlation; purple indicates positive correlation). (**H**) Analysis of KEGG pathways co-annotated by differential metabolites and differential functional genes. (**I**) Comparative significance analysis of the riboflavin metabolism pathway between the COPD and control groups. (**J**–**L**) Abundance distributions of three differential functional genes associated with riboflavin metabolism in the two groups. (* *p* < 0.05, ** *p* ≤ 0.01, *** *p* ≤ 0.001). Note: In the volcano plot, blue dots represent significantly downregulated metabolites, red dots represent significantly upregulated metabolites, and gray dots represent metabolites without significant differences. The *x*-axis indicates log_2_-transformed fold change, and the *y*-axis indicates −log_10_-transformed *p* values from *t*-tests, the horizontal dashed line indicates *p* = 0.05 (−log_10_(*p*) = 1.3), metabolites with reduced abundance in the COPD group are shown on the left of the vertical dashed line, while those with increased abundance are on the right. In the KEGG enrichment dot plot, the *x*-axis represents the Rich Factor, the *y*-axis lists pathway names, bubble size corresponds to the number of differential metabolites, and color intensity reflects enrichment significance, with darker red indicating greater significance.

Alterations in gut microbiota function can influence host physiology through changes in metabolite composition. To characterize metabolic disturbances associated with gut ecological imbalance in COPD patients, untargeted metabolomic profiling was performed on fecal samples. Both Principal component analysis (PCA) and Orthogonal partial least squares–discriminant analysis (OPLS-DA) demonstrated clear separation between the COPD and control groups (Figure 4A,B), suggesting pronounced differences in fecal metabolic profiles. Multivariate analysis identified 497 metabolites that differed significantly between groups, including 397 upregulated and 100 downregulated metabolites in patients with COPD (Figure 4C). The top 10 metabolites exhibiting the greatest fold increases and decreases are presented in Figure 4D. Notably, metabolites such as 7-phosphonovalerate and 3-hydroxy-3-methyl-2-oxobutanoic acid were significantly reduced in the COPD group, whereas ethylbenzene and 16-octylpalmitate were markedly elevated relative to healthy controls.

Kyoto Encyclopedia of Genes and Genomes (KEGG) pathway enrichment analysis of differential metabolites was performed using the clusterProfiler package with a hypergeometric test approach (Figure 4E). The most significantly enriched pathway was Phenylalanine, tyrosine and tryptophan biosynthesis, with six differential metabolites mapped to this pathway. This pathway is central to the biosynthesis of aromatic amino acids that serve not only as essential substrates for protein synthesis but also as precursors for multiple neurotransmitters and hormones, suggesting dysregulation of amino acid metabolism in COPD. Concurrently, the differential metabolite L-tyrosine, which participates in this biosynthetic pathway, was also involved in the Prolactin signaling pathway, indicating potential cross-regulation between metabolic and neuroendocrine signaling processes in COPD. In addition, Biotin metabolism was significantly enriched, involving four differential metabolites, including Biotinyl-5′-AMP and Pimelate. Given the essential role of biotin as a coenzyme for carboxylases involved in lipid and carbohydrate metabolism, perturbations in this pathway may contribute to altered energy homeostasis in COPD. Enrichment of the Diabetic cardiomyopathy pathway, which included three differential metabolites, including L-carnitine, suggests potential overlap between metabolic disturbances in COPD and cardiovascular complications. Significant changes were also observed in the Nitrogen metabolism pathway, with alterations in metabolites such as nitrous oxide and carbamate, indicating possible disruption of nitrogen balance in COPD patients (Figure 4F). Collectively, these pathway-level alterations delineate characteristic changes in the fecal metabolome of COPD patients and provide additional metabolic insight into disease pathogenesis.

### 2.10. Correlation Between Gut Microbiota and Fecal Metabolites

To further elucidate interactions between gut microbiota and fecal metabolites in COPD and to assess the potential predictive value of microbiome-related metabolites, an integrated metagenomic–metabolomic association analysis was performed. This analysis incorporated the differentially abundant microbial taxa and metabolites identified above. Significant correlations were observed between fecal metabolites and gut microbiota composition in patients with COPD (Figure 4G). By jointly mapping differential functional genes and differential metabolites to the KEGG database, four shared pathways were identified: Penicillin and cephalosporin biosynthesis, Riboflavin (RF) metabolism, Starch and sucrose metabolism, and Neomycin, kanamycin and gentamicin biosynthesis (Figure 4H). Among these pathways, Riboflavin metabolism (ko00740) exhibited a consistent downregulated trend in COPD patients (Figure 4I). Furthermore, key functional genes associated with this pathway—diaminohydroxyphosphoribosylaminopyrimidine deaminase/5-amino-6-(5-phosphoribosylamino)uracil reductase (K11752), 6,7-dimethyl-8-ribityllumazine synthase (K00794), and 3,4-dihydroxy-2-butanone 4-phosphate synthase/GTP cyclohydrolase II (K14652)—were all significantly reduced in the COPD group (Figure 4J–L). These coordinated reductions indicate a marked impairment in the riboflavin biosynthetic capacity of the gut microbiota in COPD patients.

### 2.11. Serum Metabolomic Characteristics and Functional Alterations Associated with COPD

**Figure 5:**
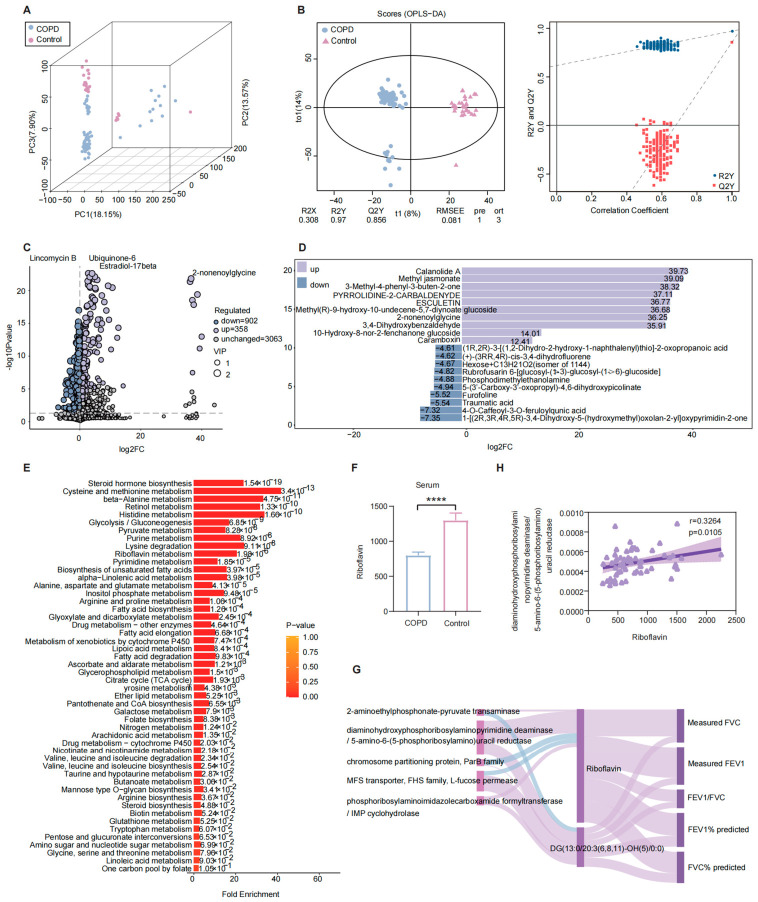
Serum metabolomic characteristics and integrated functional analyses in COPD and control groups. (**A**) PCA score plot comparing serum metabolite profiles between the COPD and control groups. (**B**) OPLS-DA score plot and corresponding permutation test plot; R^2^ indicates model goodness of fit, and Q^2^ reflects predictive performance. (**C**) Volcano plot of differential serum metabolites between the COPD and control groups, with pink indicating significantly upregulated metabolites and blue indicating significantly downregulated metabolites. (**D**) Fold change analysis of differential metabolites, showing log-transformed fold change (logFC) values for the top 10 upregulated and top 10 downregulated metabolites in the COPD group relative to controls. (**E**) MSEA plot depicting the top 25 significantly enriched metabolic pathways. (**F**) Bar plot comparing serum riboflavin abundance between the COPD and control groups. (**G**) Sankey diagram illustrating correlations among differential functional genes, differential serum metabolites, and pulmonary function indicators; functional genes are shown on the left, serum metabolites in the center, and pulmonary function indices on the right (pink denotes positive correlations; blue denotes negative correlations). (**H**) Correlation analysis between serum riboflavin levels and the functional gene *K11752*. (**** *p* ≤ 0.0001).

Given that gut microbiota–derived metabolites can enter systemic circulation and influence distal organs, untargeted metabolomic profiling of serum samples from patients with COPD and healthy controls was performed using an LC-QTOF platform. PCA demonstrated clear separation between the two groups (Figure 5A), which was further confirmed by OPLS-DA (Figure 5B). The OPLS-DA model showed strong explanatory and predictive performance (R^2^Y (cum) = 0.97, Q^2^ (cum) = 0.856), and permutation testing verified the robustness and reliability of the model. Across all serum samples, a total of 4323 metabolites were annotated, among which 1260 metabolites differed significantly between the COPD and control groups (Figure 5C). Compared with healthy controls, 358 metabolites were significantly elevated in patients with COPD, including calanolide A, methyl jasmonate, 3-methyl-4-phenyl-3-buten-2-one, pyrrolidine-2-carbaldehyde, and adenosine tetraphosphate. In contrast, 902 metabolites were significantly reduced in the COPD group, including 4-O-caffeoyl-3-O-feruloylquinic acid, traumatic acid, furofoline, riboflavin, and phosphodimethylethanolamine (Figure 5D). Metabolite Set Enrichment Analysis (MSEA) revealed significant enrichment of several metabolic pathways in the COPD group relative to controls, notably steroid hormone biosynthesis, cysteine and methionine metabolism, riboflavin metabolism, and retinol metabolism (Figure 5E). These findings indicate widespread systemic metabolic reprogramming associated with COPD.

Consistent with the metabolomic and gut microbial functional analyses, serum riboflavin levels were significantly lower in patients with COPD than in healthy controls (Figure 5F). To evaluate the clinical relevance of these metabolic alterations, correlation analyses were conducted among gut microbial functional genes, serum metabolites, and pulmonary function parameters. Serum riboflavin levels were positively correlated with measured FVC, FVC% predicted, measured FEV_1_, FEV_1_% predicted, and the FEV_1_/FVC ratio (Figure 5G), indicating that reduced riboflavin levels are closely associated with impaired lung function. Furthermore, we found that the differential functional gene K11752, which is annotated to the riboflavin metabolism pathway, exhibited a significant positive correlation with serum riboflavin concentrations (Figure 5H). This finding suggests that alterations in gut microbiota functional capacity may modulate systemic riboflavin availability through regulation of riboflavin biosynthesis, thereby contributing to the pathophysiological processes underlying COPD.

### 2.12. Identification of Potential Biomarkers for COPD Using Random Forest and ROC Analysis

**Figure 6:**
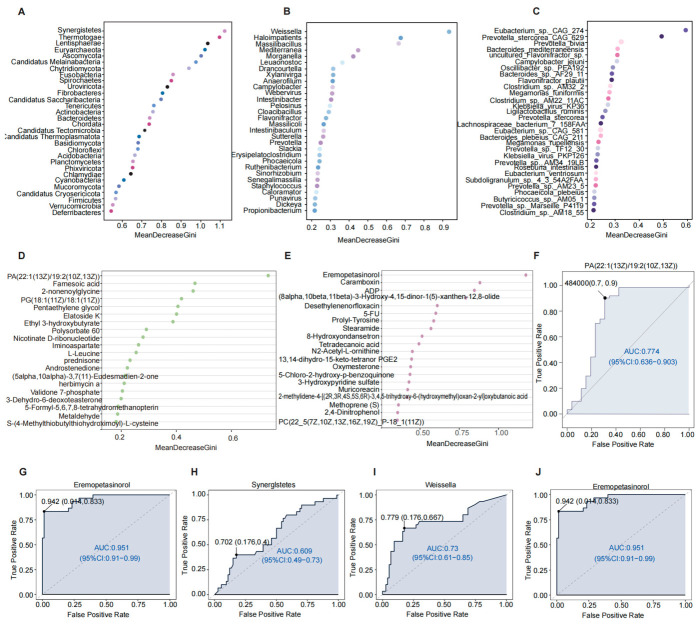
Random forest–based feature importance ranking and diagnostic performance of microbiota- and metabolite-derived markers. (**A**–**C**) Random forest importance ranking plots showing the top 30 discriminatory microbial taxa at the phylum (**A**), genus (**B**), and species (**C**) levels, based on their contribution to distinguishing COPD patients from healthy controls. (**D**,**E**) Random forest importance ranking of the top 20 discriminatory fecal metabolites (**D**) and serum metabolites (**E**) identified according to their contribution to classification performance. (**F**,**G**) ROC curve analyses evaluating the diagnostic performance of the most informative fecal (**F**) and serum (**G**) metabolites. (**H**–**J**) ROC curve analyses assessing the diagnostic performance of the most informative microbial features at the phylum (**H**), genus (**I**), and species (**J**) levels.

Alterations in the abundance of key microbial taxa can disrupt gut microbial homeostasis and may provide discriminative features for disease classification. To identify candidate biomarkers for COPD, representative core microbial taxa were selected from both COPD patients and healthy controls using random forest analysis. The top 30 taxa at each taxonomic level (phylum, class, order, family, genus, and species) were ranked according to their contribution to classification performance, as quantified by the Mean Decrease Gini (MDG) index. At the phylum level, *Synergistetes* emerged as the most informative taxon for distinguishing COPD patients from controls. At the class level, *Epsilonproteobacteria* showed the greatest contribution to classification accuracy, while *Campylobacterales* was the most influential order. At the family level, *Nitrosomonadaceae* demonstrated the highest discriminative importance. At finer taxonomic resolution, the genus *Weissella* played a prominent role in differentiating COPD patients from healthy individuals, and *Eubacterium_sp._CAG_274* was identified as the most informative species-level feature (Figure 6A–C and Figures S5–S7). In parallel, random forest analysis of metabolomic data identified PA(22:1(13Z)/19:2(10Z,13Z)) as the most informative fecal metabolite and Eremopetasinorol as the most informative serum metabolite for COPD classification (Figure 6D,E).

To evaluate diagnostic performance, receiver operating characteristic (ROC) curve analysis was applied to the most informative microbial taxa at the phylum, genus, and species levels, as well as the most informative fecal and serum metabolites. The area Under the Curve (AUC) values for Synergistetes, Weissella, and *Eubacterium*_sp._CAG_274 were 0.605, 0.735, and 0.597, respectively. The fecal metabolite PA(22:1(13Z)/19:2(10Z,13Z)) yielded an AUC of 0.774, whereas the serum metabolite eremopetasinorol demonstrated the highest diagnostic performance, with an AUC of 0.947 (Figure 6F–J).

Collectively, these findings indicate that eremopetasinorol exhibits strong discriminatory capacity and represents a promising serum biomarker for COPD. Moreover, the integrated results from random forest feature selection and ROC analysis suggest that serum metabolites provide superior diagnostic performance compared with fecal metabolites and gut microbiota–based indicators.

## 3. Discussion

In this study, integrated metagenomic and metabolomic approaches were applied to comprehensively characterize gut microbiota composition, functional potential, and metabolic profiles in 74 patients with stable COPD and 30 healthy controls from northern China. By moving beyond 16S rRNA sequencing, which is limited in taxonomic and functional resolution, this work addresses key gaps in prior studies that were constrained by small sample sizes, insufficient functional analyses, and a lack of data from Chinese populations. The results demonstrate pronounced alterations in gut microbiota structure, function, and host-associated metabolic profiles in COPD, supporting an important role for gut microbial dysregulation in disease pathophysiology. At the compositional level, LEfSe analysis identified 23 taxa with significantly different abundances between COPD patients and healthy controls (LDA ≥ 3), indicating distinct microbial signatures associated with COPD. Functional profiling further revealed enrichment of genes involved in signal transduction, altered expression of specific glycoside hydrolases, and increased abundance of antibiotic resistance genes, including *baeS*. At the metabolic level, 497 differential fecal metabolites and 1260 differential serum metabolites were identified. By combining metagenomic sequencing with serum untargeted metabolomics and fecal untargeted metabolomics, we obtained unique insights that could not be derived from any single omics dataset alone. Serum riboflavin levels were significantly reduced in COPD patients and were positively correlated with pulmonary function indices, including FEV_1_ and FVC (*p* < 0.05), as well as with the key differential functional gene diaminohydroxyphosphoribosylaminopyrimidine deaminase/5-amino-6-(5-phosphoribosylamino)uracil reductase (K11752) (*p* < 0.05). Among all candidate biomarkers, serum eremopetasinorol exhibited the highest diagnostic performance (AUC = 0.947). Collectively, these findings provide evidence supporting a regulatory role of the gut–lung axis in COPD among individuals from northern China and offer new perspectives for precision diagnosis and targeted intervention.

The gut–lung axis has emerged as a conceptual framework for elucidating the complex and bidirectional interactions between the gastrointestinal tract and pulmonary system [15]. Clinically, patients with COPD frequently exhibit comorbid chronic gastrointestinal disorders, including inflammatory bowel disease and irritable bowel syndrome [16]. These conditions are closely linked to gut microbial dysbiosis, increased intestinal permeability, and inflammatory cell infiltration [17]. Previous studies have also demonstrated that intestinal epithelial damage and compromised barrier function are present in patients with COPD, contributing to gastrointestinal functional disturbances under chronic inflammatory conditions [18]. Conversely, alterations in gut microbiota composition and microbe-derived metabolites may actively participate in COPD pathogenesis.

With respect to microbial diversity, this study observed no significant difference in gut microbiota alpha diversity between patients with COPD and healthy controls, a finding consistent with the report by Kou et al. [19]. In contrast, beta diversity analysis demonstrated a significant separation in overall gut microbiota composition between the COPD and control groups, indicating that COPD is associated with distinct alterations in microbial community structure. This observation aligns with the majority of existing studies reporting gut microbiota dysbiosis in patients with COPD [20,21] and suggests that structural remodeling of the gut microbiota may represent an important component of COPD-associated pathophysiology. Further compositional analysis revealed specific microbial shifts in COPD patients. Compared with healthy controls, COPD patients exhibited a reduced relative abundance of *Bacteroidetes* and an increased abundance of *Firmicutes* [22,23,24], consistent with previous findings and in agreement with the results of the present study. In addition, the genus *g_Subdoligranulum* was significantly enriched in COPD patients. Li et al. reported that patients with acute exacerbations of COPD are particularly susceptible to environmental exposures and identified a positive correlation between air pollutants and microbial function, specifically a positive correlation between particulate matter (PM10) levels and *Subdoligranulum* abundance [25]. These observations suggest that enrichment of *Subdoligranulum* may be associated with disease severity or environmental sensitivity in COPD, supporting the relevance of this taxon in the present findings. Notably, at the genus level, *Weissella* emerged as an important discriminator between COPD patients and healthy individuals. *Weissella* has been reported to possess probiotic-related properties [26], including the production of antimicrobial substances, competitive exclusion of pathogenic bacteria, and modulation of host immune responses [27,28]. However, its specific role in the development and progression of COPD remains unclear and warrants further investigation.

At the functional level, this study identified significant upregulation of genes involved in signal transduction mechanisms in COPD patients, along with abnormal expression of glycoside hydrolases, including GH116 and GH130. In parallel, antibiotic resistance genes such as *baeS* and *smeS* were enriched, accompanied by increased resistance to pleuromutilin and sulfonamide antibiotics. Glycoside hydrolases, including GH116, participate in the degradation of complex carbohydrates, and their dysregulated expression may disrupt intestinal energy metabolism. Given that patients with COPD frequently exhibit increased energy expenditure and nutritional imbalance, such functional alterations may further influence metabolic homeostasis through gut–lung axis–related pathways. The enrichment of antibiotic resistance genes within the gut microbiota of COPD patients is also noteworthy and may reflect long-term or repeated exposure to antibiotic therapies. Previous studies have demonstrated that the annual frequency of antibiotic use is significantly higher in individuals with COPD than in healthy populations [29]. Antibiotic exposure can perturb gut microbial balance and promote the accumulation of resistance genes, potentially exacerbating immune dysregulation and inflammatory responses and contributing to a self-perpetuating cycle [30]. These findings underscore the importance of optimizing antibiotic treatment strategies in COPD, including consideration of resistance gene profiles to guide personalized therapy, with the aim of limiting resistance gene dissemination and reducing therapeutic complexity.

In addition, metabolomic analyses revealed pronounced disturbances in both fecal and serum metabolic profiles in patients with COPD. In fecal samples, 497 differential metabolites were identified, involving multiple metabolic pathways, including amino acid and lipid metabolism. In serum, 1260 metabolites exhibited significant alterations, encompassing key compounds such as riboflavin, calanolide A, and methyl jasmonate. MSEA demonstrated that these differential metabolites were predominantly enriched in biologically relevant pathways, notably riboflavin metabolism, cysteine and methionine metabolism, and histidine metabolism. Among these pathways, the association between RF and COPD has attracted increasing attention. Riboflavin, also known as vitamin B2, is a water-soluble vitamin with high thermal stability [31] and is indispensable for cellular metabolic processes [32]. Patients with COPD typically exhibit impaired antioxidant capacity, resulting in excessive production of reactive oxygen species (ROS), increased oxidative stress, and subsequent exacerbation of pulmonary inflammation and tissue injury. Riboflavin possesses intrinsic antioxidant properties and contributes to ROS neutralization through its role as a precursor of flavin mononucleotide (FMN) and flavin adenine dinucleotide (FAD), which serve as essential coenzymes for numerous enzymes involved in oxidative metabolism, including glutathione peroxidase (GPx). Toyasaki et al. demonstrated that glutathione reductase requires RF to catalyze the reduction of oxidized glutathione (GSSG) to reduced glutathione (GSH), a critical endogenous antioxidant that inactivates ROS. Through GPx-mediated reactions, glutathione is converted from GSH to GSSG during the detoxification of lipid peroxides, yielding alcohols and mitigating oxidative damage [33]. Accordingly, RF may alleviate oxidative stress by inhibiting lipid peroxidation and attenuating reperfusion-related oxidative injury. Given that oxidative stress is a central pathogenic mechanism in COPD, riboflavin-mediated maintenance of the glutathione redox cycle represents a critical antioxidant defense pathway in lung tissue [34,35]. Beyond its antioxidant role, riboflavin has also been implicated in the modulation of inflammatory responses. A recent study demonstrated that riboflavin attenuates *NLRP3*, *NLRC4*, *AIM2*, and non-canonical inflammasome activation by inhibiting mitochondrial ROS production and mitochondrial DNA release, thereby blocking caspase-1 activation and subsequent maturation of IL-1β and IL-18. Given the well-established involvement of the IL-1β/IL-18 axis in COPD airway inflammation and emphysema, this mechanism provides a direct link between riboflavin metabolism and COPD pathogenesis [36]. Studies by Verdrengh et al. reported that riboflavin supplementation reduced inflammatory cell infiltration, decreased neutrophil accumulation in lung tissue, attenuated inflammatory responses, and improved respiratory function in COPD patients [37]. Moreover, clinically, patients with COPD have been reported to exhibit lower riboflavin status compared with individuals without COPD, as measured by the erythrocyte glutathione reductase activation coefficient [38]. In vivo experimental studies have further validated the protective effects of riboflavin against pulmonary injury. In three distinct rat models of oxidant-mediated acute lung injury, riboflavin administration significantly reduced vascular permeability (by 31–56%), alveolar hemorrhage (by 51–76%), and lipid peroxidation products (by 45% in lung tissue) [39]. Collectively, these findings suggest that riboflavin may exert multifaceted protective effects in the pathogenesis and progression of COPD.

Humans are unable to synthesize riboflavin endogenously and therefore depend on dietary intake, intestinal absorption, and microbial biosynthesis within the gut. Notably, approximately 40% of riboflavin can be produced by the gut microbiota. A systematic genomic analysis of B-vitamin biosynthesis pathways demonstrated that complete riboflavin operons are present in the genomes of all *Bacteroidetes* and *Fusobacteria*, as well as in 36 genomes (accounting for 92%) of *Proteobacteria*, with de novo riboflavin synthesis pathways identified in nearly all members of these phyla. In contrast, within *Actinobacteria,* only two publicly available genomes—*Corynebacterium ammoniagenes* DSM 20306 and *Bifidobacterium longum* ATCC 15697—possess the genetic capacity for riboflavin biosynthesis [40]. George et al. further demonstrated that *Escherichia coli* harbors a complete riboflavin biosynthesis operon (rib operon) and can efficiently synthesize riboflavin under aerobic conditions [41]. However, in patients with COPD, this biosynthetic capacity may be markedly reduced as a result of intestinal congestion and associated hypoxic conditions. Together, these observations indicate that gut microbiota represent a critical exogenous source of riboflavin and that their biosynthetic capacity is strongly influenced by both microbial community structure and host physiological status.

To elucidate the molecular mechanisms underlying the impaired riboflavin biosynthetic capacity of the gut microbiota in patients with COPD, the present study conducted a focused analysis of the riboflavin metabolism pathway. The results demonstrated a significant downregulation of this pathway in COPD patients. Consistently, three key functional genes annotated to riboflavin biosynthesis—K11752, K00794, and K14652—were all significantly reduced in abundance in COPD patients. The enzymes encoded by these genes play essential roles in the microbial riboflavin pathway. K11752, also known as RibD, is a critical bifunctional enzyme that catalyzes an early “deamination–reduction” step in riboflavin biosynthesis, converting an unstable pyrimidine precursor into a structurally stable intermediate that forms the foundation of the pathway [42]. K00794 (RibH) catalyzes the condensation of 5-amino-6-ribitylamino-2,4(1H,3H)-pyrimidinedione with 3,4-dihydroxy-2-butanone 4-phosphate to generate the pteridine derivative DMRL, which serves as the sole substrate for downstream riboflavin synthase [43]. K14652, designated RibA [44], uniquely integrates two rate-limiting enzymatic activities—GTP cyclohydrolase II and DHBP synthase—within a single polypeptide, thereby coordinating substrate flux across parallel branches of the pathway and enabling efficient regulation of overall riboflavin production [45]. The coordinated downregulation of the riboflavin metabolism pathway and its core biosynthetic genes provides a molecular basis for the reduced riboflavin synthesis capacity of the gut microbiota in COPD, ultimately diminishing microbial contribution to host riboflavin supply.

In parallel, riboflavin availability in humans also depends on dietary intake and intestinal absorption. Riboflavin is primarily obtained from animal-derived foods and fortified grains, with comparatively lower levels present in plant-based sources. Intestinal uptake is mediated by riboflavin transporters (RFVTs) expressed in the small intestinal mucosa [46]. Subramanian et al. demonstrated that mice with targeted knockout of RFVT3 developed severe riboflavin malabsorption, highlighting the essential role of these transporters in maintaining riboflavin homeostasis [47]. In patients with COPD, the presence of cor pulmonale frequently leads to intestinal congestion, resulting in mucosal ischemia and hypoxia. Chronic hypoxic conditions can suppress RFVT2 transcription through hypoxia-inducible factor–mediated pathways, while inflammatory mediators further inhibit RFVT expression [46], collectively reducing riboflavin absorption efficiency. Supporting the reversibility of this process, Gariballa et al. reported that riboflavin supplementation at a dose of 10 mg/day for 8 weeks increased plasma riboflavin concentrations to 312 ± 58 nmol/L, approaching levels observed in healthy individuals, while reducing fecal riboflavin excretion to 157 ± 41 µg, indicating partial restoration of intestinal absorption capacity [38]. These findings provide a mechanistic rationale for riboflavin supplementation in COPD patients. Taken together, insufficient dietary intake or impaired absorption, combined with reduced microbial biosynthetic capacity, likely contributes to the observed decline in serum riboflavin levels in COPD patients.

Importantly, integrated metagenomic and untargeted metabolomic analyses revealed a previously unrecognized link between gut microbial dysfunction, systemic metabolic alterations, and impaired pulmonary function in COPD. The key microbial gene K11752 showed a strong association with serum riboflavin concentrations, and serum riboflavin levels were positively correlated with lung function indices. These findings support the existence of a gut microbiota–riboflavin metabolism–lung function regulatory axis. This framework not only offers a mechanistic explanation for riboflavin deficiency in COPD but also identifies modulation of gut microbial riboflavin biosynthesis as a potential therapeutic avenue to improve riboflavin metabolism and support disease management in COPD.

The identification of reliable biomarkers is crucial for the early diagnosis and longitudinal monitoring of COPD. In this study, random forest analysis combined with ROC curve evaluation identified the serum metabolite eremopetasinorol as exhibiting the strongest diagnostic performance for COPD (AUC = 0.947, 95% CI: 0.88–0.98), markedly outperforming fecal metabolites and gut microbiota–based indicators. Compared with microbiome-derived markers, serum biomarkers are generally less susceptible to inter-individual variability and environmental influences, thereby offering greater stability and reproducibility in clinical applications. Eremopetasinorol is an exceptionally rare metabolite; research on this metabolite is still in its preliminary stages, and available evidence regarding its biological origin and physiological function remains limited. To infer the potential origin of Eremopetasinorol, we performed a comprehensive cross-database analysis. Specifically, BioDeep explicitly classified it as a human metabolite, an endogenous natural product, while HMDB noted its presence in dietary sources such as green vegetables but provided no evidence for microbial production. To date, only a single study suggests that alterations in the metabolic abundance of Eremopetasinorol are associated with early-life in vivo exposure to perfluorooctane sulfonate (PFOS), and that PFOS exposure may induce liver inflammation by disrupting gut-liver crosstalk [48]. While the exact mechanism of Eremopetasinorol in COPD remains to be elucidated, its structural classification as an eremophilane-type sesquiterpenoid—a family known for anti-inflammatory and immunomodulatory activities [49]—suggests potential involvement in the inflammatory pathways central to COPD pathogenesis. Future studies should further explore its potential mechanisms of action in patients with COPD and validate its suitability as a serum biomarker for COPD.

Our findings have several potential clinical implications. First, the reduced serum riboflavin levels and their positive correlation with lung function suggest that riboflavin supplementation could serve as a low-cost adjunctive strategy to alleviate oxidative stress in COPD, though optimal dosage and patient subgroups require further investigation in randomized controlled trials. Second, altered gut microbial taxa (e.g., Weissella) and functional genes (e.g., K11752 in riboflavin metabolism) raise the possibility of probiotic or prebiotic approaches aimed at restoring beneficial gut bacteria and enhancing microbial riboflavin biosynthesis. Third, serum eremopetasinorol demonstrated high diagnostic accuracy (AUC = 0.947), outperforming fecal metabolites and gut microbial features. Although this rare metabolite requires further biological characterization, its strong discriminatory performance supports future large-scale validation as a non-invasive early diagnostic biomarker for COPD.

Several limitations of this study should be acknowledged. First, the absence of long-term follow-up restricts the ability to comprehensively evaluate the sustained effects of riboflavin on disease progression. Second, metabolic and microbiome-related outcomes may vary across populations, and the influence of environmental exposures, dietary patterns, and regional factors warrants careful consideration. Specifically, the general dietary pattern in northern China is relatively homogeneous, characterized by wheat-based staples and coarse grains as the main sources of carbohydrates, along with relatively fixed patterns of vegetable, meat, and fermented food consumption [50]. Such dietary patterns are known to influence the composition of gut microbiota, including Prevotella [51,52]. All participants in this study were recruited from the same northern city, had long-term local residence, and shared broadly similar dietary habits; therefore, the confounding effect of dietary factors on intergroup comparisons is relatively small. Nevertheless, individual-level dietary data were not collected in this study, and the potential impact of dietary differences on the results cannot be completely excluded. Consequently, the generalizability of these findings to genetically diverse populations with different lifestyles and dietary habits may be limited. Third, although this study only enrolled patients receiving inhaled corticosteroids and bronchodilators and excluded those with recent use of systemic corticosteroids or antibiotics, we did not perform subgroup analyses based on the type or dose of inhaled medications. Therefore, the potential impact of inhaled medications on the gut microbiota and metabolic profiles cannot be completely ruled out. In addition, the associations observed in this study have not yet been validated in animal models or interventional clinical trials. Finally, as this was an observational rather than an interventional study, it was not possible to fully control for all factors that may influence gut microbiota composition and COPD disease status. This study is captures a single time-point snapshot and cannot infer causality or track intra-individual dynamics of the microbiota and metabolome during acute exacerbations or disease progression. While we strictly controlled sampling time to reduce temporal heterogeneity, true longitudinal tracking is required. Future research should therefore prioritize large-scale, multicenter, prospective longitudinal cohort, incorporating standardized dietary assessment tools, to further validate the relationships between gut microbiota, microbial metabolites, and COPD. Moreover, in-depth investigations examining the differential effects of RF across diverse ethnicities, age groups, and sex are needed to enhance the generalizability and clinical relevance of these findings.

## 4. Materials and Methods

### 4.1. Study Population and Evaluation Criteria

Participants were recruited between June 2023 and May 2024 from the Department of Respiratory and Critical Care Medicine at Beijing Chaoyang Hospital, Capital Medical University (ClinicalTrials.gov registration number: NCT03044847). In total, 74 patients with COPD and 30 healthy controls were enrolled. Eligibility criteria for the COPD group included: (1) age > 40 years; (2) fulfillment of the GOLD 2021 diagnostic criteria, defined as a post-bronchodilator FEV_1_/FVC < 70% of the predicted value; (3) clinical stability with no acute exacerbations within the preceding 4 weeks; and (4) a documented history of smoking (Smoking index ≥ 10 packs/year). Healthy controls were defined as individuals with normal pulmonary function confirmed by spirometry and no history of chronic respiratory disease. Exclusion criteria applied to both groups were as follows: (1) coexisting asthma, active pulmonary tuberculosis, interstitial pneumonia, or severe bronchiectasis; (2) severe comorbidities (acute infection, diabetes mellitus, stroke, cardiovascular disease, hepatic or renal insufficiency, malignancy, or autoimmune diseases); (3) a history of chronic diarrhea or constipation; (4) prior gastrointestinal surgery; (5) hyperlipidemia; (6) use of probiotics or antibiotics within 4 weeks prior to enrollment; (7) use of oral corticosteroids or Chinese herbal medicine within the preceding 3 months; and (8) pregnancy or lactation. All enrolled patients with COPD were in a stable disease state at the time of inclusion. Comprehensive demographic and clinical data were collected, including exposure to indoor and outdoor environmental pollutants, duration of COPD diagnosis, smoking status, medication use, dietary habits, and GOLD classification. Blood sampling and pulmonary function testing were performed for all participants. The study protocol was approved by the Ethics Committee of Beijing Chaoyang Hospital, Capital Medical University (Approval No. 2023-5-25-6), and written informed consent was obtained from all participants prior to enrollment.

### 4.2. Sample Collection and Biochemical Index Assessment

Peripheral blood samples were collected from healthy individuals and patients with COPD. Following standardized preprocessing procedures, serum was isolated by centrifugation at 3000 rpm for 10 min, and the supernatant was collected. The centrifugation speed and time were strictly controlled to avoid hemolysis. Biochemical indices were measured using a fully automated biochemical analyzer. Remaining serum samples were stored at −80 °C until further analysis. Hemolyzed serum samples were discarded to ensure the quality of metabolomic analysis. Fresh fecal samples (approximately 6 g) were collected from each participant, with material obtained from the central portion of the stool to minimize environmental contamination. Samples were immediately aliquoted into sterile cryotubes (approximately 3 g per tube) and stored at −80 °C for subsequent analyses.

### 4.3. Metagenomic Analysis

Total fecal DNA was extracted using the cetyltrimethylammonium bromide method according to standard protocols, and three technical replicates were set for each sample to ensure extraction reproducibility. DNA concentration was quantified using a Qubit 3.0 Fluorometer (Invitrogen, Qubit™ dsDNA HS Assay Kit), and DNA integrity was assessed by 1% agarose gel electrophoresis. For library preparation, 10 ng of high-quality genomic DNA, as quantified by Qubit, was transferred into a 96-well plate and adjusted to the required volume with nuclease-free water. The DNA then underwent enzymatic fragmentation, end repair, ligation product purification, PCR amplification, and size selection of amplified fragments. Library fragment quality was assessed using the Qsep-400 system, and library concentration was re-quantified using the Qubit 3.0 Fluorometer. Prepared libraries were sequenced on an Illumina NovaSeq 6000 platform using the NovaSeq 6000 S4 Reagent Kit (Illumina, San Diego, CA, USA). The raw reads obtained from the above sequencing contained low-quality sequences.

### 4.4. Bioinformatics Analysis

Raw sequencing reads were initially quality-filtered using Trimmomatic to generate high-quality clean reads. Host-derived sequences were subsequently removed by aligning the filtered reads to the human reference genome using Bowtie2. De novo metagenomic assembly was performed using MEGAHIT (version 1.1.2) with default parameters, and assembled contigs shorter than 300 bp were excluded from further analysis. Assembly quality was evaluated using QUAST (version 2.3) with default settings. Open reading frames were predicted from assembled contigs using MetaGeneMark (version 3.26; parameters: −A −D −f G). To reduce redundancy, predicted gene sequences were clustered using MMseqs2, applying a sequence identity threshold of 95% and a coverage threshold of 90% to generate a non-redundant gene catalog. Protein sequences derived from this catalog were aligned against the NCBI non-redundant (Nr) protein database using DIAMOND BLAST (version 0.9.29.130), with an E-value cutoff of ≤1 × 10^−5^. For each gene, functional annotation was assigned based on the best-matching reference sequence. Functional annotation and abundance profiling were conducted by aligning non-redundant protein sequences against the KEGG, eggNOG, CAZy, and CARD databases. Differentially abundant taxonomic features between the COPD and control groups were identified using LEfSe, with a LDA score threshold set at ≥3.0. The raw metagenomic sequencing data have been deposited in the NCBI Sequence Read Archive (SRA) under the BioProject accession number PRJNA1454801.

### 4.5. Metabolomic Analysis

Metabolites were extracted from fecal and serum samples using methanol-based protein precipitation, with three technical replicates per sample. A quality control (QC) sample was prepared by mixing equal volumes of all samples to monitor the stability of the extraction process. Untargeted metabolomic profiling was performed using a liquid chromatography–mass spectrometry (LC–MS) platform comprising a Waters Acquity I-Class PLUS ultra-performance liquid chromatography (UPLC) system coupled to a Waters Xevo G2-XS quadrupole time-of-flight (QTOF) high-resolution mass spectrometer. Chromatographic separation was achieved using an Acquity UPLC HSS T3 column (1.8 µm, 2.1 mm × 100 mm). The Xevo G2-XS QTOF mass spectrometer was operated in MSe acquisition mode under the control of MassLynx version 4.2 software (Waters), enabling simultaneous collection of precursor (MS1) and fragment ion (MS2) spectra. Raw LC–MS data were processed using Progenesis QI software (version 4.0) for peak detection, retention time alignment, normalization, and data filtering. Metabolite identification was performed by matching accurate mass, retention time, and fragmentation patterns against the online METLIN database, publicly available metabolite databases, and a curated in-house database integrated within Progenesis QI, with additional confirmation based on theoretical fragment ion matching. After database searching, overall data quality was further assessed by intra-group reproducibility (evaluated by PCA clustering) and inter-group differences (greater separation in PCA and lower inter-group correlation indicate stronger group discrimination). The fecal non-targeted metabolomics data and serum non-targeted metabolomics data have been deposited in the MetaboLights database under the accession numbers MTBLS14336 and MTBLS14338, respectively.

### 4.6. Statistical Analysis

All statistical analyses were performed using the R programming environment. Within-sample (alpha) diversity was assessed by calculating the Shannon and Simpson indices, and differences between the COPD and control groups were evaluated using Welch’s t-test. Between-sample (beta) diversity was quantified using Bray–Curtis distance matrices and visualized by PCoA. Group-level differences in beta diversity were statistically tested using analysis of similarities (ANOSIM). Differentially abundant taxonomic features between groups were identified using LEfSe. For functional analysis, the non-redundant gene catalog was annotated by comparison against functional gene and metabolic databases, including KEGG, eggNOG, CAZy, and CARD, using the DIAMOND alignment tool. Differences in functional module abundance between groups were evaluated using non-parametric statistical tests. Correlations between microbial species were assessed using Spearman’s rank correlation coefficient.

In untargeted metabolomics analyses, PCA and Spearman correlation analysis were applied to assess sample repeatability within groups and to evaluate the stability of quality control samples. Identified metabolites were annotated with classification and pathway information by querying the KEGG, HMDB, and LipidMaps databases. Group-wise fold changes were calculated for each metabolite, and statistical significance was determined using *t*-tests. OPLS-DA was conducted using the R language package *ropls*, and model robustness was assessed through 200 permutation tests. Variable importance in projection (VIP) scores were calculated using cross-validation. Differential metabolites were identified by integrating fold change, *p* value, and VIP score, with screening criteria defined as FC > 1, *p* value < 0.05, and VIP > 1. KEGG pathway enrichment analysis of differential metabolites was performed using the hypergeometric distribution test.

To integrate metagenomic and metabolomic datasets, Spearman correlation analysis was employed to examine associations between differentially abundant microbial species, functional genes, and differential metabolites. Correlations between differential metabolites and differential functional genes were further analyzed using R. Random forest analysis combined with ROC curve analysis was used to identify candidate biomarkers from microbial and metabolic profiles and to evaluate their diagnostic performance.

## 5. Conclusions

Using an integrated metagenomic and metabolomic framework, this study delineated significant alterations in both the composition and functional capacity of the gut microbiota in patients with COPD. A total of 23 differentially abundant microbial taxa were identified, accompanied by functional perturbations involving signal transduction pathways, glycoside hydrolase activity, and antibiotic resistance gene profiles. Importantly, this work provides the first evidence that gut microbial dysfunction may contribute to reduced serum riboflavin levels through disruption of riboflavin metabolism, a change that is closely associated with impaired pulmonary function. Viewed within the context of the gut–lung axis, these findings offer novel insights into the pathological mechanisms underlying COPD. In parallel, the identification of candidate metabolic biomarkers highlights potential avenues for earlier diagnosis and targeted intervention. Further validation through large-scale clinical studies and mechanistic investigations in animal models is required to confirm these observations and to clarify their biological and clinical significance.

## Figures and Tables

**Table 1:** Patient characteristics.

	COPD (n = 74)	Control (n = 30)	*p* Value
Gender, Male, n (%)	61 (82.43)	21 (70.00)	<0.0001
Age (year), mean ± SD	67.81 ± 7.75	67.87 ± 3.88	0.1662
BMI (kg/m^2^), mean ± SD	24.95 ± 3.55	25.37 ± 3.03	0.0185
Smoking history (pack-years), median ± IQR	22.50 ± 30.94	0.00 ± 1.38	<0.0001
Measured FVC, mean ± SD	2.88 ± 0.72	3.38 ± 0.66	<0.0001
FVC% predicted, mean ± SD	88.14 ± 16.88	99.3 ± 13.03	0.0227
Measured FEV_1_, mean ± SD	1.41 ± 0.51	2.67 ± 0.49	<0.0001
FEV1% predicted, mean ± SD	56.24 ± 19.35	96.8 ± 12.94	<0.0001
FEVI/FVC, mean ± SD	58.51 ± 15.20	79.45 ± 5.33	<0.0001

Abbreviations: SD: Standard Deviation; BMI: Body Mass Index; IQR: Interquartile Range.

## Data Availability

The raw metagenomic sequencing datasets for all samples have been deposited in the Sequence Read Archive (SRA) database under the accession number PRJNA1454801. The fecal non-targeted metabolomics data and serum non-targeted metabolomics data have been deposited in the MetaboLights database under the accession numbers MTBLS14336 and MTBLS14338. The authors declare that all data supporting the findings of this study are available within the paper or from the corresponding authors upon request.

## Supplementary Materials

The following supporting information can be downloaded at: https://www.mdpi.com/article/10.3390/ijms27104213/s1.

## Abbreviations

Abbreviation	Full name
COPD	Chronic obstructive pulmonary disease
RF	Riboflavin
LEfSe	Linear discriminant analysis effect size
ROC	Receiver operating characteristic
SCFAs	Short-chain fatty acids
GOLD	Chronic Obstructive Lung Disease
KEGG	Kyoto Encyclopedia of Genes and Genomes
eggNOG	Evolutionary genealogy of genes: Non-supervised Orthologous Groups
CAZy	Carbohydrate-Active enZYmes
CARD	Comprehensive Antibiotic Resistance Database
LDA	Linear discriminant analysis
LC–MS	Liquid chromatography–mass spectrometry
UPLC	Ultra-performance liquid chromatography
QTOF	Quadrupole time-of-flight
PCoA	Principal coordinates analysis
ANOSIM	Analysis of similarities
PCA	Principal component analysis
OPLS-DA	Orthogonal partial least squares–discriminant analysis
VIP	Variable importance in projection
SD	Standard Deviation
BMI	Body Mass Index
IQR	Interquartile Range
PerMANOVA	Permutational multivariate analysis of variance
ARGs	Antibiotic resistance genes
MSEA	Metabolite Set Enrichment Analysis
MDG	Mean Decrease Gini
ROS	Reactive oxygen species
FMN	Flavin mononucleotide
FAD	Flavin adenine dinucleotide
GPx	Glutathione peroxidase
GSSG	Oxidized glutathione
GSH	Reduced glutathione
RFVTs	Riboflavin transporters
PFOS	Perfluorooctane sulfonate
AUC	Area Under the Curve
SRA	Sequence Read Archive
QC	Quality control
