# Supplementary figures and images for: Characteristics of Gut Microbiota in Patients with Chronic Obstructive Pulmonary Disease Based on Metagenomics and Metabolomics
Source: Int J Mol Sci. 2026 May 9;27(10):4213. doi: 10.3390/ijms27104213 (PMC13207120; doi:10.3390/ijms27104213)

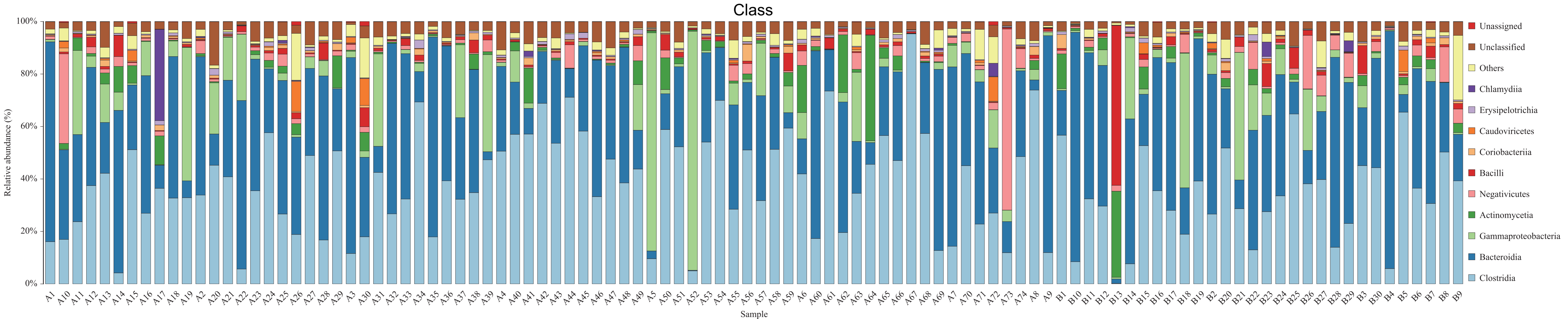

Supplement: Supplementary file 1 [file ijms-27-04213-s001.zip › Supplementary Figure S1.pdf]

Order

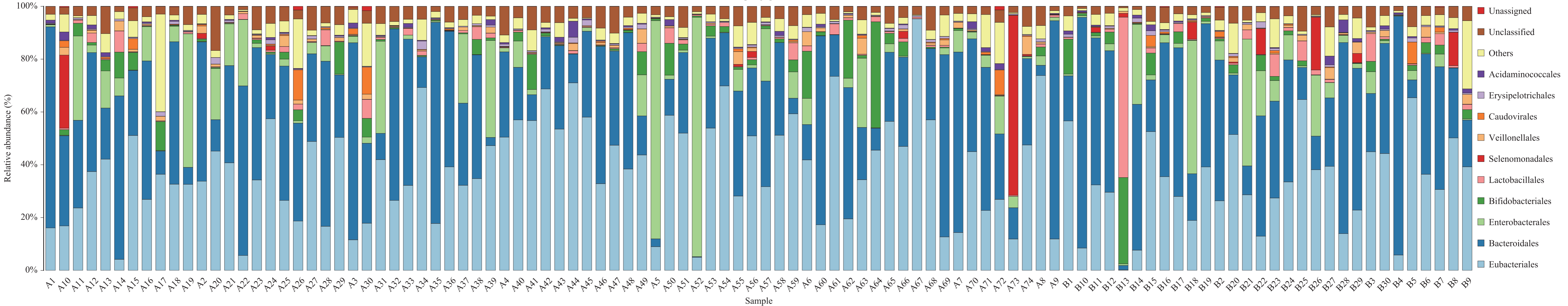

Supplement: Supplementary file 1 [file ijms-27-04213-s001.zip › Supplementary Figure S2.pdf]

Family

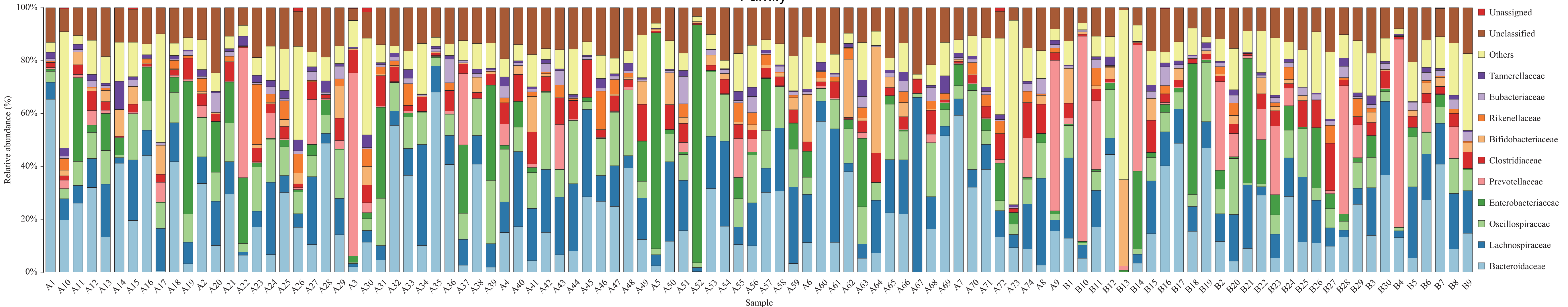

Supplement: Supplementary file 1 [file ijms-27-04213-s001.zip › Supplementary Figure S3.pdf]

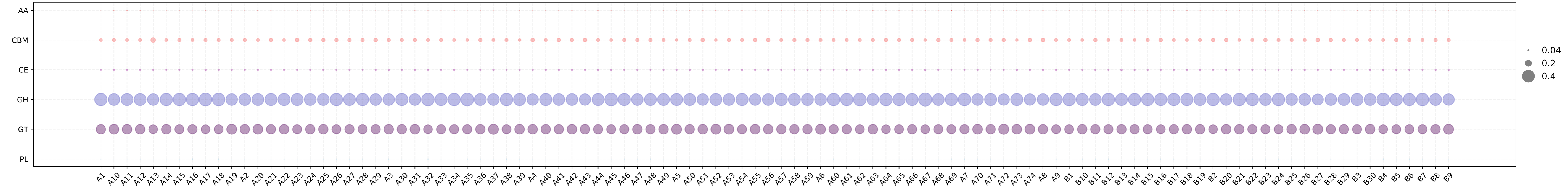

Supplement: Supplementary file 1 [file ijms-27-04213-s001.zip › Supplementary Figure S4.pdf]

# Class

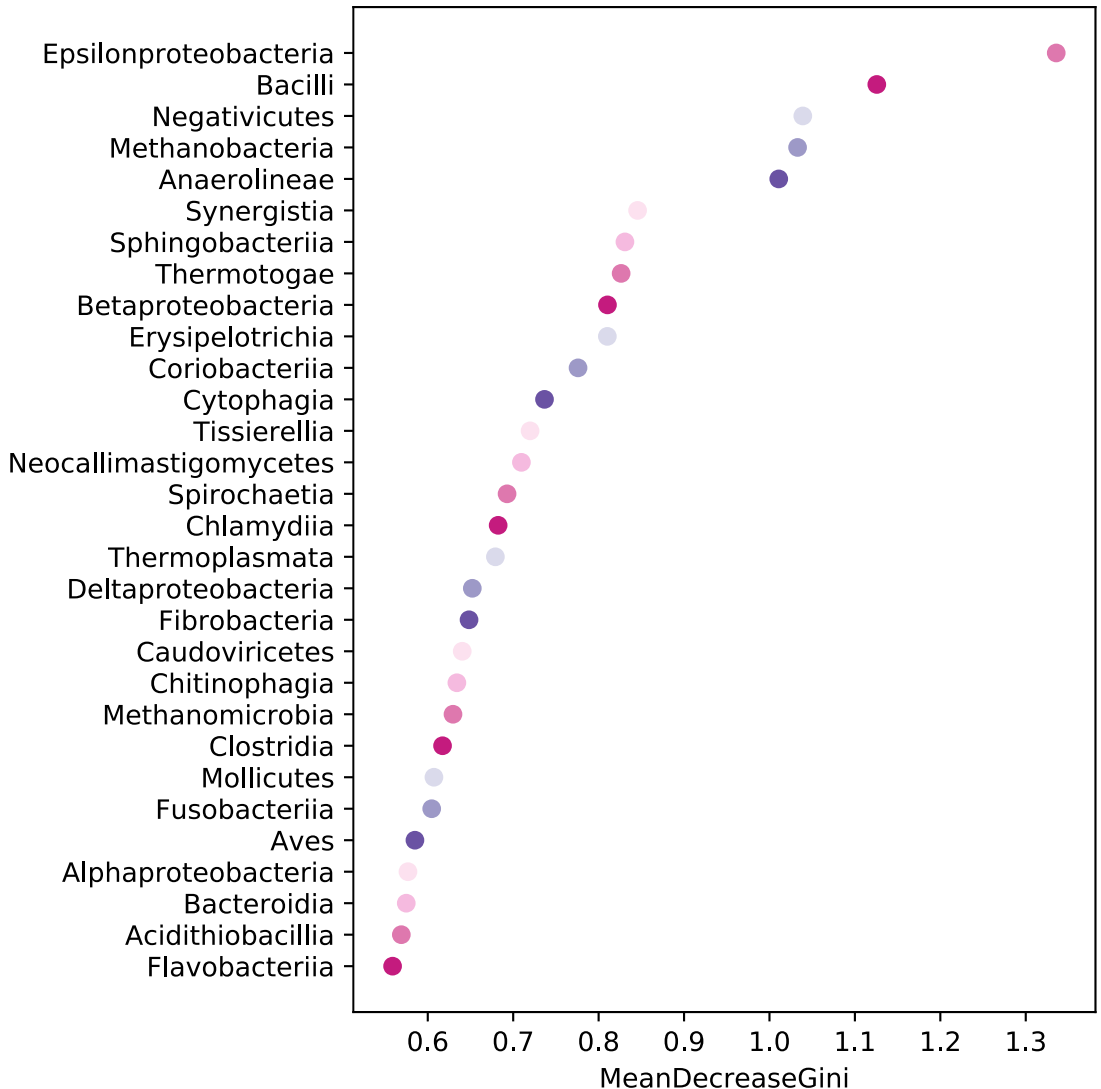

Supplement: Supplementary file 1 [file ijms-27-04213-s001.zip › Supplementary Figure S5.pdf]

# Order

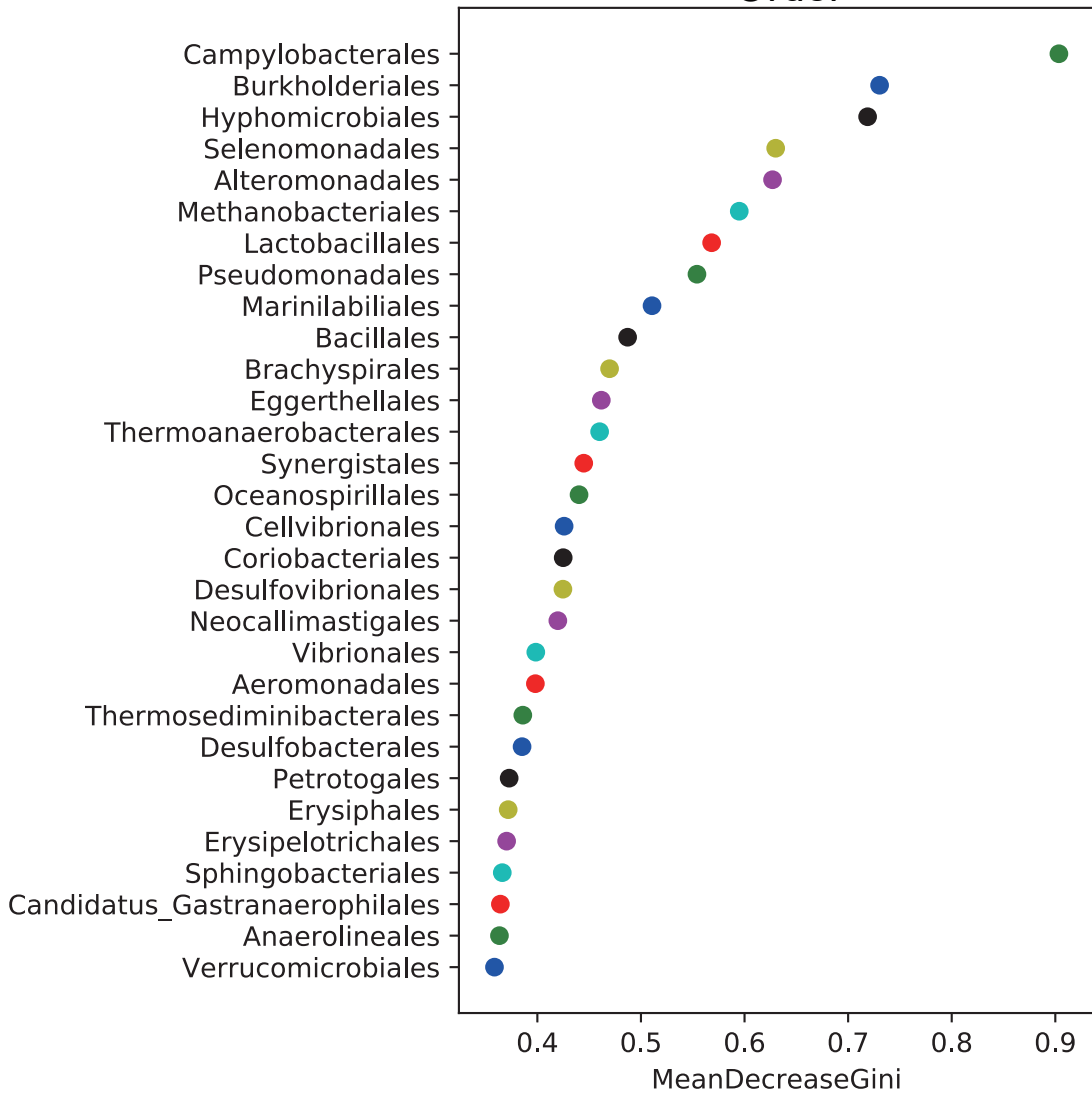

Supplement: Supplementary file 1 [file ijms-27-04213-s001.zip › Supplementary Figure S6.pdf]

# Family

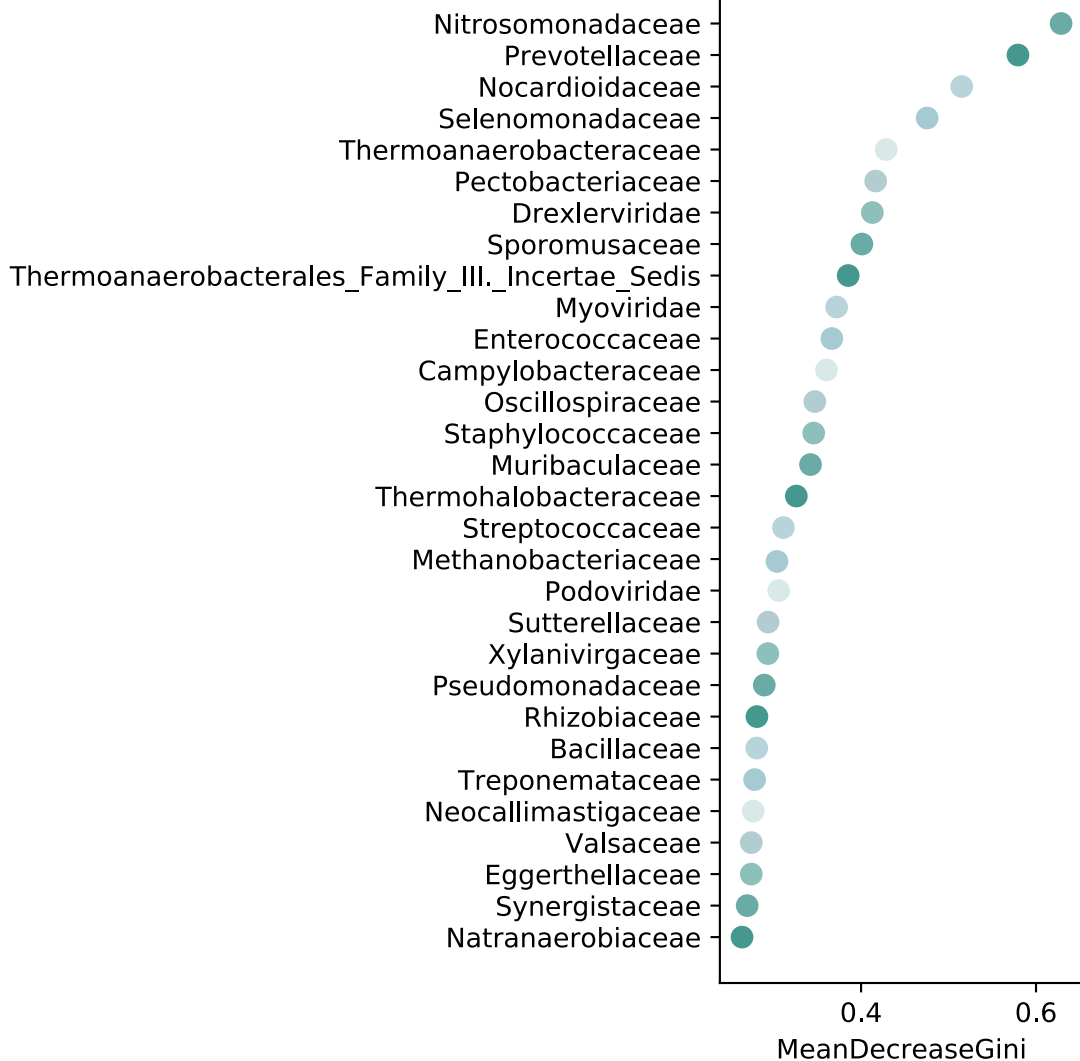

Supplement: Supplementary file 1 [file ijms-27-04213-s001.zip › Supplementary Figure S7.pdf]
